# Supplementary material for: Nuclear Beclin 1 Destabilizes Retinoblastoma Protein to Promote Cell Cycle Progression and Colorectal Cancer Growth
Source: Cancers (Basel). 2022 Sep 28;14(19):4735. doi: 10.3390/cancers14194735 (PMC9563141; doi:10.3390/cancers14194735)
Supplement: Supplementary file 1 [file cancers-14-04735-s001.zip › cancers-1849763-Figure and Table S1.pdf]

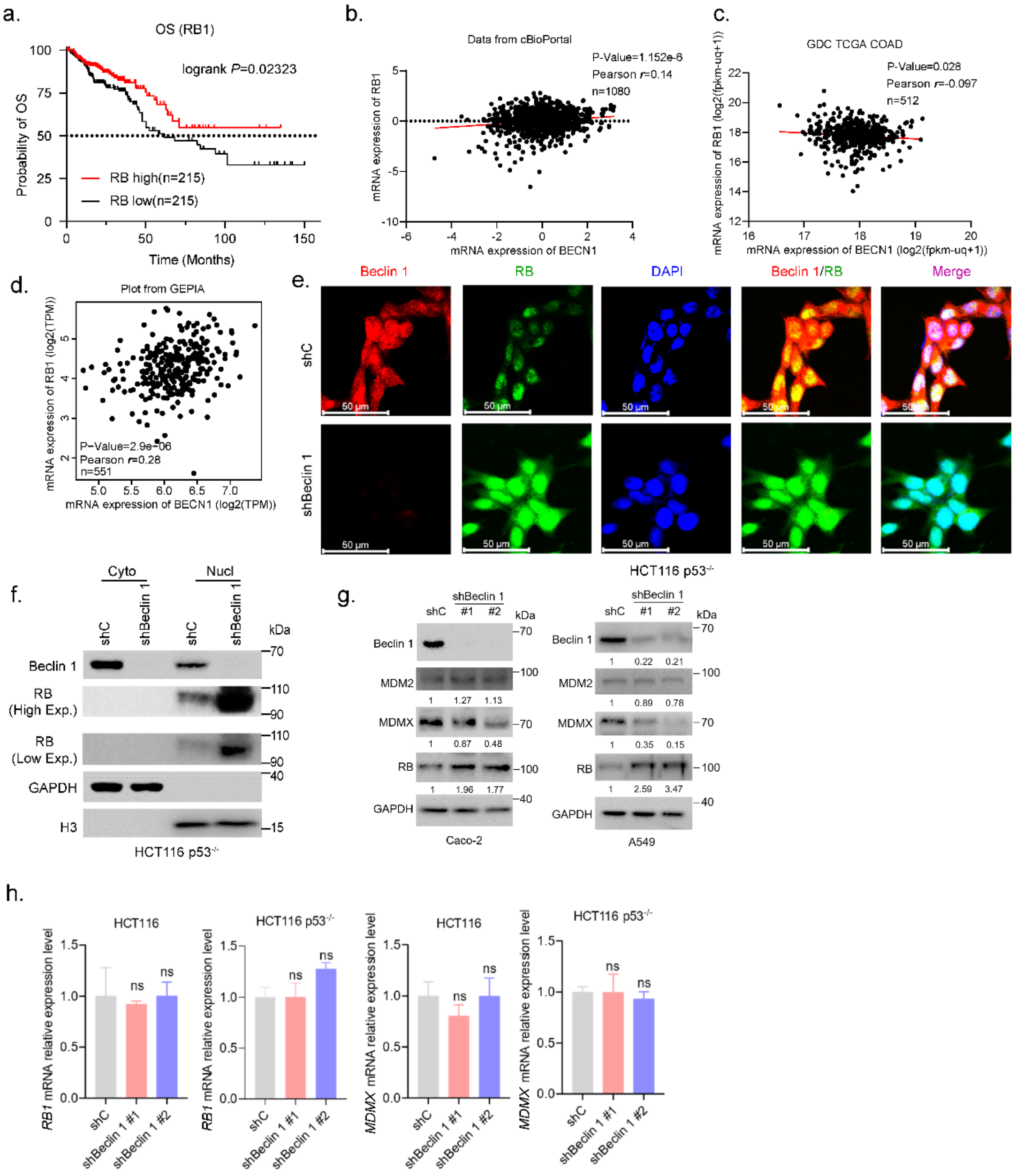

Supplementary Figure S1. Beclin 1 is co-localized with RB in nuclei. Knockdown of *BECN1* has no significant effect on the steady-state *RB1* mRNA levels.

a. Low *RB1* expression is associated with poor overall survival of human colorectal cancer patients. Kaplan-Meier plots of overall survival (OS) of human colorectal cancer patients were stratified by the *RB1* mRNA expression levels obtained from the TCGA COADREAD dataset (access on 3 May 2022).

b-d. There was no significant correlation between *BECN1* and *RB1* mRNA levels in colon cancer patient samples. Pearson correlation analyses were conducted between *BECN1* and *RB1* mRNA levels in colon cancer patient samples. **(b)** The mRNA expression datasets include TCGA Firehose Legace (access on 10 September 2022), TCGA PanCancer Atlas and CPTAC-2 Prospective from cBioPortal (access on 10 September 2022). The gene expression data were transformed to z-scores relative to all samples based on log2(RSEM) values in each dataset. **(c)** Data from GDC TCGA COAD. The gene expression data were expressed as log2(fpkm-uq+1) (access on 10 September 2022. **(d)** Data from GEPIA. (access on 10 September 2022) The gene expression data were expressed as log2(TPM). Transcripts Per Million, TPM; Fragments Per Kilobase of exon Per million read, FPKM; RNA-Seq by Expectation-Maximization, RSEM.

e-f. Knockdown of *BECN1* upregulates RB protein expression in nuclei.

(e) HCT116 $p53^{-/-}$ cells stably expressing shC or shBeclin 1 were subjected to immunofluorescent (IF) staining for endogenous Beclin 1 (red) or RB (green) and counter-staining nuclei with DAPI (blue). Scale bar=50 μm. **(f)** Total cell lysates derived from HCT116 $p53^{-/-}$ cells stably expressing shC or shBeclin 1 were subjected to a nuclear-cytoplasmic fractionation assay.

g. Caco-2 and A549 cells stably expressing shC or shBeclin 1 were subjected to western blot.

h. HCT116 or HCT116 $p53^{-/-}$ cells stably expressing shC or shBeclin 1 (#1 or #2) were subjected to qPCR analyses for *RB1 or MDMX* mRNA levels, *GAPDH* was used as an internal control. Three independent experiments were performed in triplicates. ns: not significant.

Supplement Table S1.

qPCR primers

Gene name	Primer sequences
Human *BECN1*	F-AGGTTGAGAAAGGCGAGACA<br>R-GCTTTTGTCCACTGCTCCTC
Human *GAPDH*	F-CCCGTGGAATGGAATGAGATT<br>R-CTCAGGAGAGGAGCCATTT
Human *MDMX*	F-GCAAGAAATTTAACTCTCCAAGCAA<br>R-CTTTGAACAATCTGAATACCAATCCTT
Human *MDM2*	F-TTGGCGTGCCAAGCTTCTCT<br>R-TACCTGAGTCCGATGATTCC
Human *RB1*	F-GACCCAGAAGCCATTGAAATCT<br>R-GGTGTGCTGGAAAAGGGTCC

Short-hairpin RNA oligo sequences.

Name	Oligo sequences
pLKO.1-shGFP	TCCTAAGGTTAAGTCGCCCTCG
pLKO.1-shBeclin 1#1	CCCGTGGAATGGAATGAGATT
pLKO.1-shBeclin 1#2	CTCAGGAGAGGAGCCATTT
pLKO.1-shRB#1	TTTGGACTAGAAATAATGTGG
pLKO.1-shRB#2	ATCGCTGTGATCCAATTTCG

Small interfering RNA oligo sequences.

Name	Sense chain (5’-3’)
hMDM2	GCCTGGCTCTGTGTGTAAT
hMDMX	CACCTAGAAGTAATGGCTCAA
shScramble (target hMDMX)	ATAACTAGGAGGCAACACTCT
shScramble (target hMDM2)	ATGCTCGGTCCATGTGTTG
