# Supplementary material for: Nuclear Beclin 1 Destabilizes Retinoblastoma Protein to Promote Cell Cycle Progression and Colorectal Cancer Growth
Source: Cancers (Basel). 2022 Sep 28;14(19):4735. doi: 10.3390/cancers14194735 (PMC9563141; doi:10.3390/cancers14194735)
Supplement: Supplementary file 1 [file cancers-14-04735-s001.zip › cancers-1849763-File S1-WB raw data.pptx]

## Slide 1
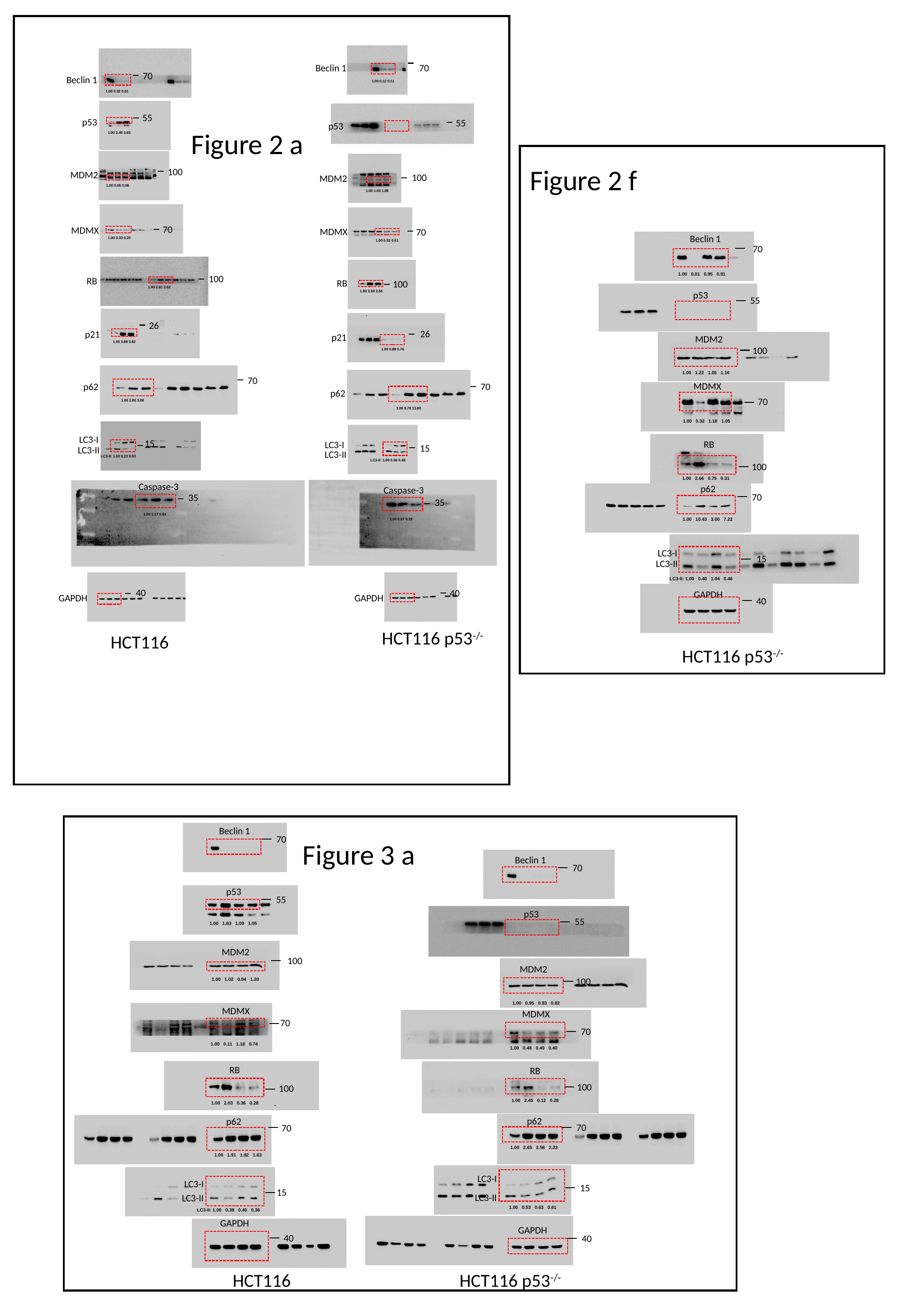

70
Beclin 1
70
Beclin 1
1.00 0.12 0.11
1.00 0.02 0.01
55
p53
55
p53
Figure 2 a
1.00 3.49 3.65
100
MDM2
100
MDM2
Figure 2 f
1.00 0.95 0.98
1.00 1.03 1.08
70
MDMX
MDMX
70
Beclin 1
1.00 0.33 0.20
1.00 0.52 0.51
70
100
RB
RB
100
1.00 0.01 0.95 0.91
1.00 2.81 2.62
1.00 3.59 3.56
p53
55
26
p21
26
p21
MDM2
1.00 3.88 3.62
100
1.00 0.88 0.76
70
p62
1.00 1.22 1.05 1.16
MDMX
70
p62
70
1.00 2.80 3.08
1.00 9.70 13.80
1.00 0.32 1.18 1.05
LC3-I
15
LC3-II
LC3-I
15
LC3-II
RB
LC3-II: 1.00 0.23 0.03
LC3-II: 1.00 0.56 0.48
100
1.00 2.66 0.75 0.31
Caspase-3
p62
Caspase-3
70
35
35
1.00 1.17 0.94
1.00 10.43 3.00 7.22
1.00 0.97 0.28
LC3-I
15
LC3-II
40
GAPDH
40
GAPDH
LC3-II: 1.00 0.40 1.04 0.46
GAPDH
40
HCT116 p53-/-
HCT116
HCT116 p53-/-
Beclin 1
70
Figure 3 a
Beclin 1
70
p53
55
p53
55
1.00 1.83 1.09 1.05
MDM2
100
MDM2
100
1.00 1.02 0.94 1.20
1.00 0.95 0.93 0.82
MDMX
MDMX
70
70
1.00 0.11 1.18 0.74
1.00 0.48 0.49 0.40
RB
RB
100
100
1.00 2.45 0.12 0.28
1.00 2.03 0.36 0.28
p62
p62
70
70
1.00 2.65 2.58 2.23
1.00 1.91 1.82 1.63
LC3-I
LC3-I
15
15
LC3-II
LC3-II
1.00 0.53 0.63 0.61
LC3-II: 1.00 0.38 0.40 0.36
GAPDH
GAPDH
40
40
HCT116 p53-/-
HCT116

## Slide 2
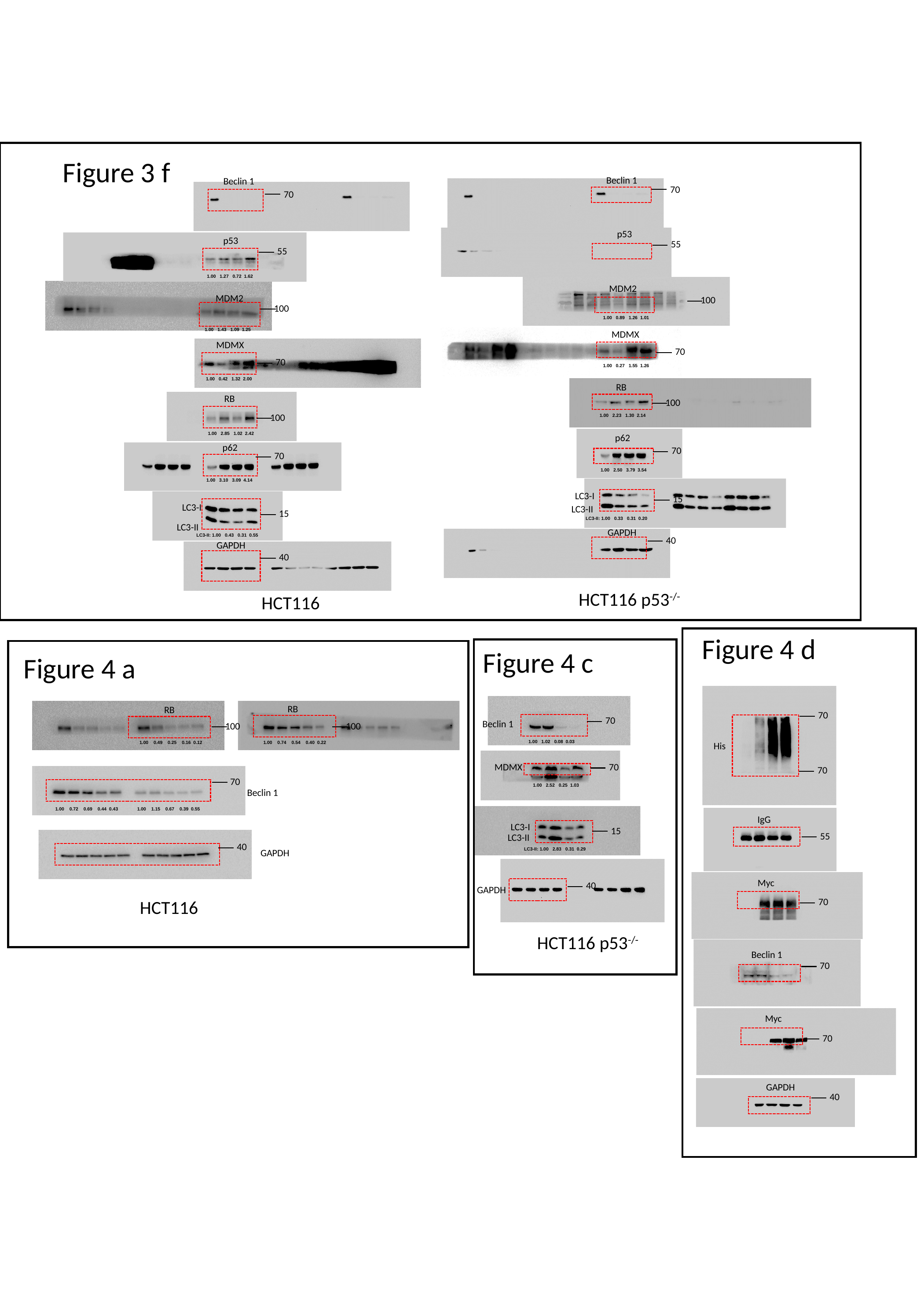

Figure 3 f
Beclin 1
Beclin 1
70
70
p53
p53
55
55
1.00 1.27 0.72 1.62
MDM2
MDM2
100
100
1.00 0.89 1.26 1.01
1.00 1.43 1.09 1.25
MDMX
MDMX
70
70
1.00 0.27 1.55 1.26
1.00 0.42 1.32 2.00
RB
RB
100
100
1.00 2.23 1.30 2.14
1.00 2.85 1.02 2.42
p62
p62
70
70
1.00 2.50 3.79 3.54
1.00 3.10 3.09 4.14
LC3-I
15
LC3-I
LC3-II
15
LC3-II: 1.00 0.33 0.31 0.20
LC3-II
GAPDH
LC3-II: 1.00 0.43 0.31 0.55
40
GAPDH
40
HCT116 p53-/-
HCT116
Figure 4 d
Figure 4 c
Figure 4 a
RB
RB
70
70
Beclin 1
100
100
1.00 1.02 0.08 0.03
1.00 0.49 0.25 0.16 0.12
1.00 0.74 0.54 0.40 0.22
His
MDMX
70
70
70
1.00 2.52 0.25 1.03
Beclin 1
1.00 0.72 0.69 0.44 0.43
1.00 1.15 0.67 0.39 0.55
IgG
LC3-I
15
55
LC3-II
40
LC3-II: 1.00 2.83 0.31 0.29
GAPDH
Myc
40
GAPDH
HCT116
70
HCT116 p53-/-
Beclin 1
70
Myc
70
GAPDH
40

## Slide 3
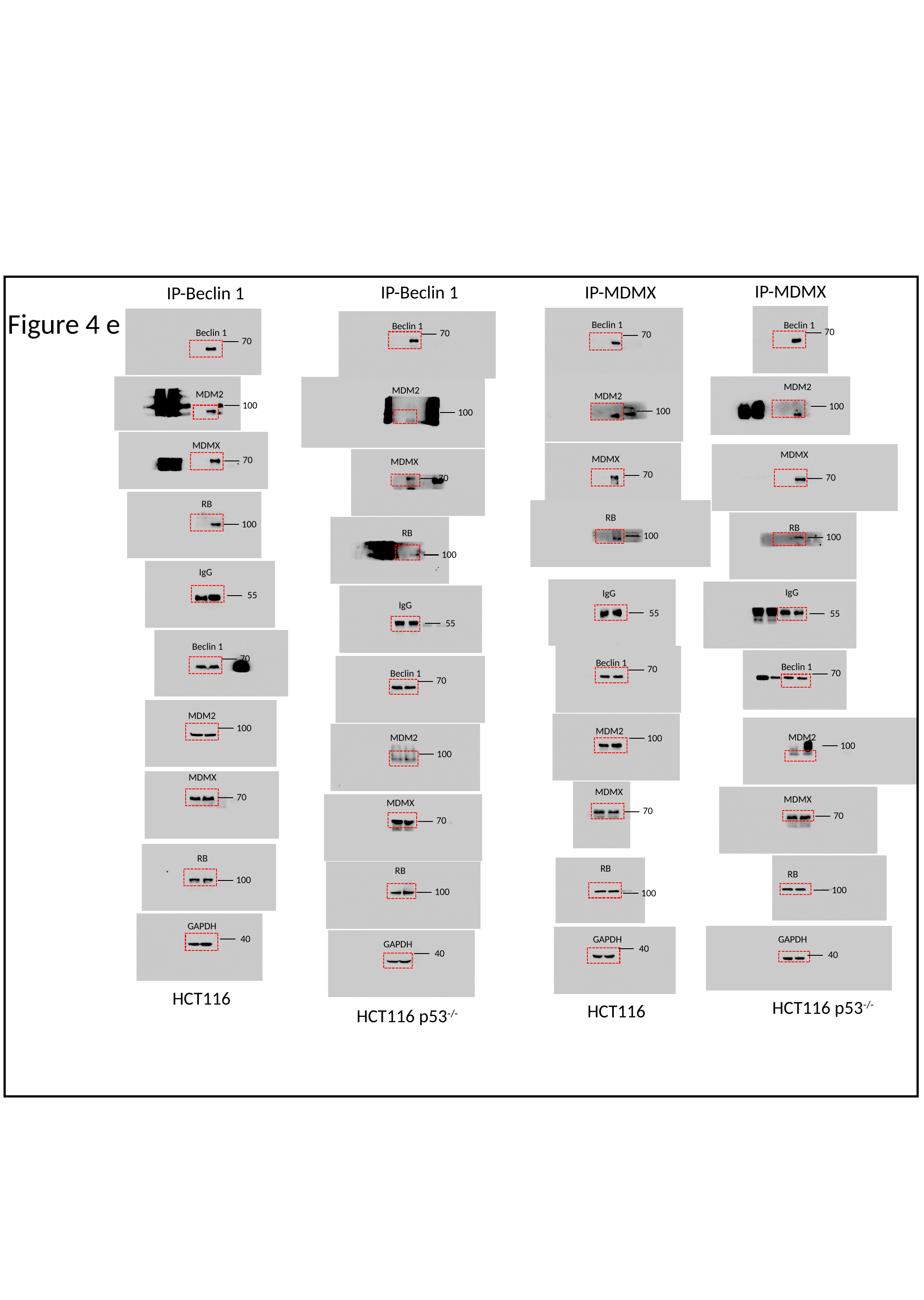

IP-MDMX
IP-MDMX
IP-Beclin 1
IP-Beclin 1
Figure 4 e
Beclin 1
Beclin 1
Beclin 1
70
Beclin 1
70
70
70
MDM2
MDM2
MDM2
MDM2
100
100
100
100
MDMX
MDMX
MDMX
70
MDMX
70
70
70
RB
RB
100
RB
RB
100
100
100
IgG
IgG
IgG
55
IgG
55
55
55
Beclin 1
70
Beclin 1
Beclin 1
70
70
Beclin 1
70
MDM2
100
MDM2
MDM2
MDM2
100
100
100
MDMX
MDMX
70
MDMX
MDMX
70
70
70
RB
RB
RB
RB
100
100
100
100
GAPDH
40
GAPDH
GAPDH
GAPDH
40
40
40
HCT116
HCT116 p53-/-
HCT116
HCT116 p53-/-

## Slide 4
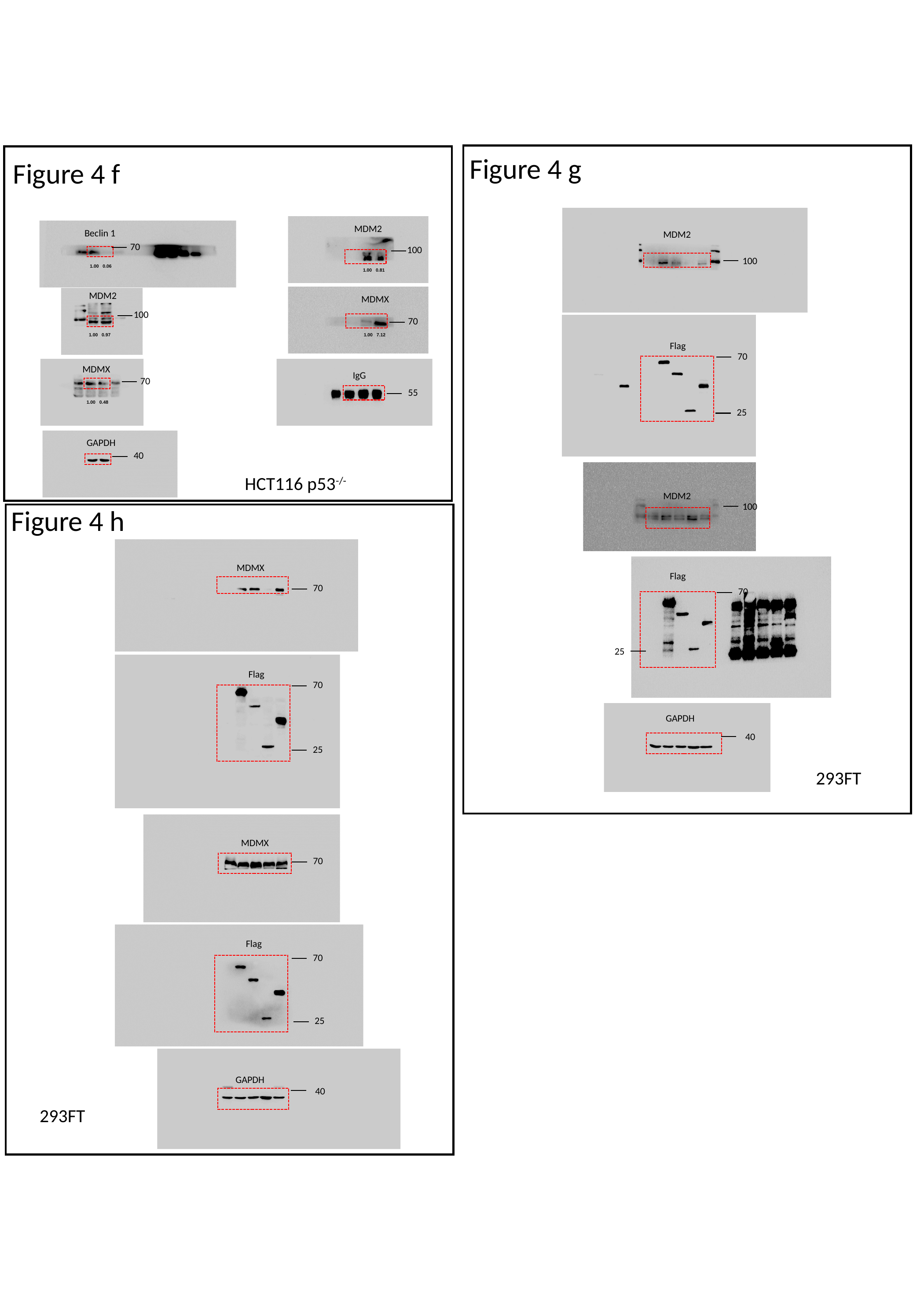

Figure 4 g
Figure 4 f
MDM2
Beclin 1
MDM2
70
100
100
1.00 0.06
1.00 0.81
MDM2
MDMX
100
70
1.00 0.97
1.00 7.12
Flag
70
MDMX
IgG
70
55
1.00 0.48
25
GAPDH
40
HCT116 p53-/-
MDM2
100
Figure 4 h
MDMX
Flag
70
70
25
Flag
70
GAPDH
40
25
293FT
MDMX
70
Flag
70
25
GAPDH
40
293FT

## Slide 5
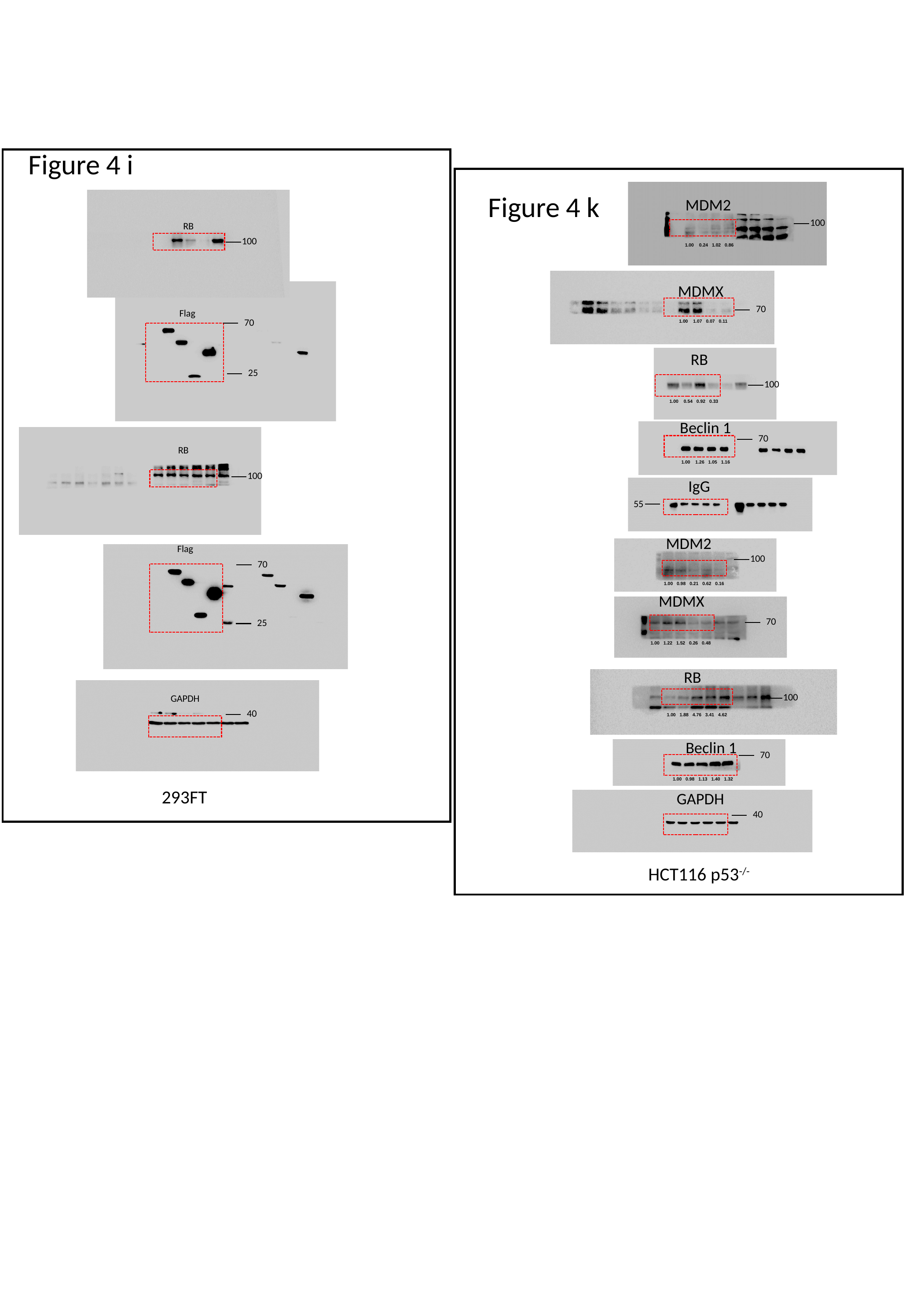

Figure 4 i
Figure 4 k
MDM2
100
RB
100
1.00 0.24 1.02 0.86
MDMX
70
Flag
70
1.00 1.07 0.07 0.11
RB
25
100
1.00 0.54 0.92 0.33
Beclin 1
70
RB
1.00 1.26 1.05 1.16
100
IgG
55
MDM2
Flag
100
70
1.00 0.98 0.21 0.62 0.16
MDMX
70
25
1.00 1.22 1.52 0.26 0.48
RB
100
GAPDH
40
1.00 1.88 4.76 3.41 4.62
Beclin 1
70
1.00 0.98 1.13 1.40 1.32
293FT
GAPDH
40
HCT116 p53-/-

## Slide 6
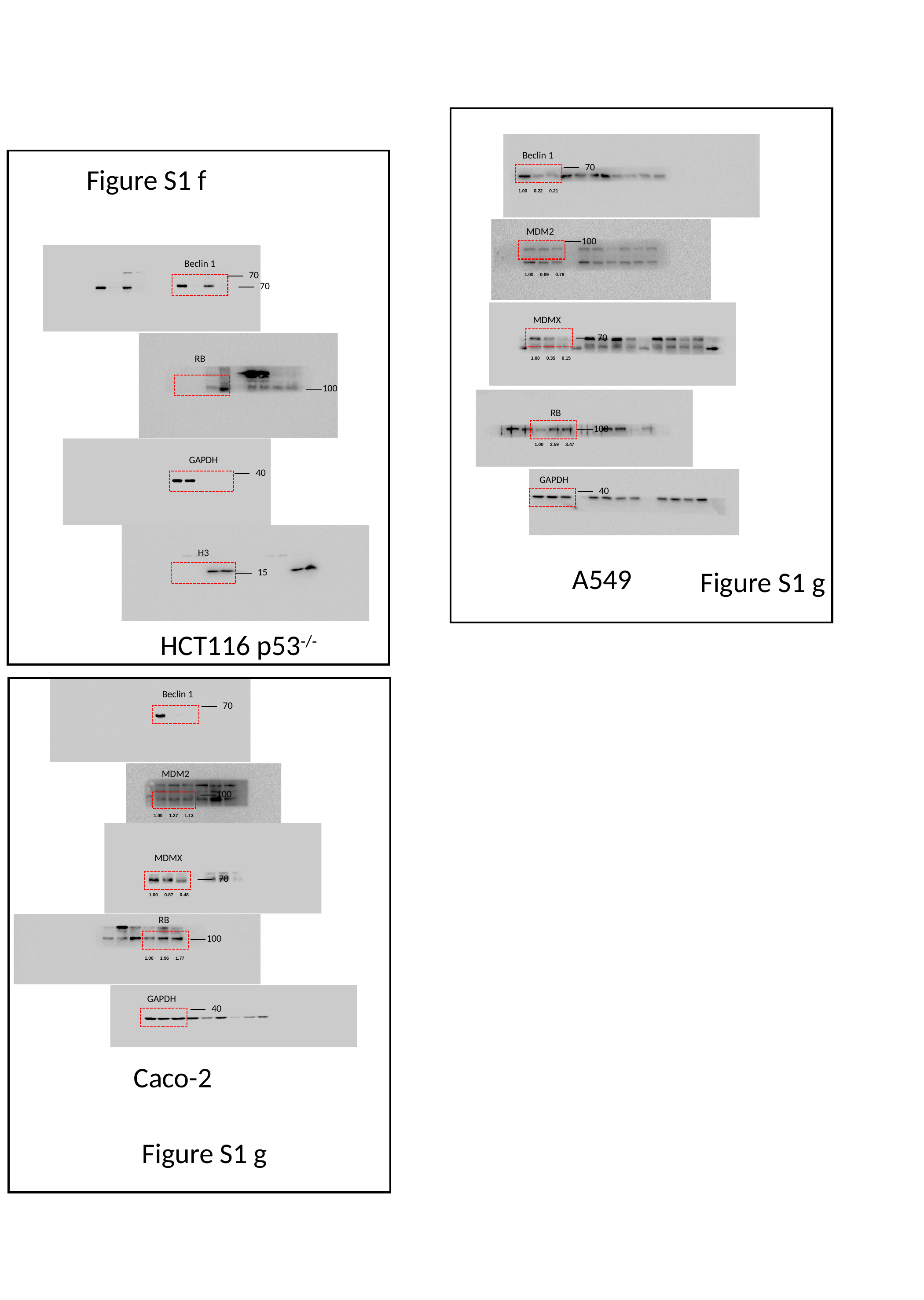

Beclin 1
70
Figure S1 f
1.00 0.22 0.21
MDM2
100
Beclin 1
70
1.00 0.89 0.78
70
MDMX
70
RB
1.00 0.35 0.15
100
RB
100
1.00 2.59 3.47
GAPDH
40
GAPDH
40
H3
A549
Figure S1 g
15
HCT116 p53-/-
Beclin 1
70
MDM2
100
1.00 1.27 1.13
MDMX
70
1.00 0.87 0.48
RB
100
1.00 1.96 1.77
GAPDH
40
Caco-2
Figure S1 g
